# The simplified method for quantifying metabolic syndrome (siMS) score reflects an increased cardiometabolic burden: adiposity, hemodynamics, and HRV findings in young adults

**DOI:** 10.1007/s40200-026-01870-2

**Published:** 2026-02-19

**Authors:** Christopher J. Kotarsky, Jillian M. Lang, Elena S. Shostak, William Quinn, Valerie Chervinskaya, Elisa Fioraso, Everett Smith, Justin A. DeBlauw, Jennifer Lloyd, Stephen J. Ives

**Affiliations:** 1 Department of Rehabilitation, Exercise and Nutrition Sciences, University of Cincinnati, 3225 Eden Avenue, Cincinnati, OH 45267 USA; 2 Department of Health and Human Physiological Sciences, Skidmore College, Saratoga Springs, NY USA; 3 Department of Biomedicine and Movement Science, University of Verona, Verona, Italy; 4 Oxford College of Emory University, Oxford, GA USA; 5 Department of Kinesiology and Outdoor Recreation, Southern Utah University, Cedar City, UT USA; 6 Optum Home Infusion Services, Eden Prairie, MN USA

**Keywords:** Metabolic syndrome, Pre-diabetes, Heart rate variability, Autonomic function, Microvascular reactivity

## Abstract

**Purpose:**

Metabolic Syndrome (MetS) increases cardiovascular disease risk, yet early identification of pre-MetS remains challenging. The Simple Method for Quantifying MetS (siMS) score offers potential for detecting cardiometabolic burden in younger and middle-aged, relatively healthy populations.

**Methods:**

We stratified 51 healthy adults (aged 18–60 years) into Low siMS (LMS, siMS < 2.085, *n* = 25) and High siMS (HMS, siMS ≥ 2.085, *n* = 26) groups based on the median siMS score and compared central hemodynamic parameters, heart rate variability (HRV), and microvascular function as independent predictors of cardiometabolic risk between these groups. Anthropometrics, body composition, hemodynamics (e.g., central blood pressure [BP], mean arterial pressure [MAP]), HRV (e.g., %R-R intervals > 50 msec, pNN50; high-frequency power), microvascular reactivity (near-infrared spectroscopy vascular occlusion test), blood glucose and lipid profiles, and dietary intake were assessed using established protocols.

**Results:**

The HMS group exhibited significantly higher body mass, body mass index (BMI), waist and hip circumferences, fat mass, visceral fat, central and peripheral BP, MAP, and augmentation index adjusted to 75 bpm (all *p* ≤ 0.004), alongside lower HRV metrics (pNN50, high-frequency power, low-frequency power, and total power, *p* ≤ 0.003), indicating increased cardiometabolic burden. No differences were observed in blood glucose, lipids, dietary intakes, or microvascular reactivity.

**Conclusion:**

These findings suggest that higher siMS scores reflect early cardiometabolic risk through adverse hemodynamic and autonomic profiles, even in the absence of overt MetS, metabolic abnormalities, or detectable microvascular dysfunction. The siMS score may serve as a proactive tool for identifying at-risk individuals, supporting targeted interventions to mitigate long-term cardiometabolic risk.

## Introduction

Metabolic syndrome (MetS), characterized by a cluster of risk factors including abdominal obesity, elevated blood pressure, dyslipidemia, high triglycerides, and hyperglycemia, significantly increases the risk of cardiovascular disease (CVD), a leading global cause of morbidity and mortality [1–4]. Early identification of MetS risk factors is critical, particularly in younger adults where these factors may be subclinical but set a trajectory for progression into adulthood, leading to adverse cardiovascular outcomes [5–10]. The Simple Method for Quantifying Metabolic Syndrome (siMS) score, derived from waist circumference, triglycerides, high-density lipoprotein (HDL) cholesterol, systolic blood pressure (SBP), and blood glucose, offers a continuous measure of MetS risk, making it a practical tool for detecting cardiometabolic burden in individuals who do not yet meet traditional MetS criteria [11, 12]. Notably, the siMS score has been associated with long-term risks of coronary heart disease, myocardial infarction, CVD, and all-cause mortality, highlighting its potential as an early screening tool for pre-MetS individuals [6, 10–12].

Central hemodynamic parameters, such as central BP, are robust predictors of CVD risk, independent of peripheral blood pressure, as they directly reflect arterial stiffness, wave reflections, and cardiovascular load [13–16]. Elevated central SBP, often driven or confounded by MetS components like visceral obesity and hypertension, is closely linked to end-organ damage and cardiovascular events, making it a critical marker for early risk assessment [13, 15–19]. Specific to MetS, previous large-scale analyses of MetS patients documented greater odds of higher central SBP, independent of sex, age, and peripheral BP, suggesting assessment of central BP is warranted [20]. Though less is known about those not yet presenting with overt MetS. Similarly, heart rate variability (HRV), a non-invasive measure of autonomic nervous system activity, provides insight into parasympathetic and sympathetic balance, with reduced HRV indicating autonomic dysfunction and heightened CVD risk [21–25]. Components of MetS, such as obesity and hypertension, may impair HRV, further exacerbating cardiovascular strain [26–28]. Additionally, microvascular dysfunction, an early marker of CVD, may contribute to cardiometabolic risk through impaired oxygen delivery and vascular reactivity, potentially, influenced by MetS risk factors [29, 30]. Assessing microvascular function using techniques like near-infrared spectroscopy vascular occlusion test (NIRS-VOT) could provide further insight into early cardiovascular changes [29, 30]. This highlights the need for research into how early risk factors, including those captured by the siMS score, influence these key cardiovascular markers, not captured by siMS, in younger and middle-aged populations.

Given that no prior studies, to our knowledge, have explored the association between the siMS score, central hemodynamic parameters, and HRV in individuals with varying siMS scores, particularly in younger, relatively healthy cohorts, this study offers a pioneering opportunity to advance our understanding of early cardiometabolic risk. The inclusion of microvascular function assessment further broadens this investigation, addressing a potential gap in understanding the full spectrum of pre-MetS risk. Investigating this area is crucial for enhancing the application of the siMS score as a proactive tool for identifying early cardiometabolic risk, especially in pre-MetS individuals, where traditional metabolic abnormalities may not yet be evident. Understanding whether higher siMS scores reflect an increased cardiometabolic burden, as indicated by central blood pressure, HRV, and microvascular function, could inform tailored preventive strategies to reduce long-term CVD risk.

The purpose of this study was to compare cardiometabolic risk in individuals stratified into low siMS score (LMS) and high siMS score (HMS) groups, focusing on hemodynamic parameters such as central blood pressure and autonomic nervous system activity assessed by HRV metrics. We also aimed to evaluate microvascular reactivity using NIRS-VOT to determine if it varies with siMS score. Given the siMS score’s association with long-term CVD risk in the absence of overt MetS, we aimed to determine whether HMS reflect an increased cardiometabolic burden, as indicated by independent CVD risk markers like central blood pressure, HRV, and microvascular function, even in a relatively healthy cohort.

## Methods

### Participants

Fifty-one healthy adults aged 18–60 years were recruited via email and flyers from Skidmore College and the surrounding community for a clinical trial registered with ClinicalTrials.gov (NCT06544915, Registration Date: 08/06/2024). Participants were screened using a self-reported health-history questionnaire to exclude those with uncontrolled chronic diseases (e.g., cardiovascular, metabolic, or pulmonary disease), two or more known cardiovascular disease (CVD) risk factors (e.g., significant smoking history), recent blood donation (< 8 weeks), cancer or current cancer treatment, pregnancy, breastfeeding, attempting to conceive, amenorrhea, hypothyroidism treated with levothyroxine, severe illness, compromised immune systems, eating disorders, or current smoking. Post-measurement, participants were stratified using the Simple Method for Quantifying Metabolic Syndrome (siMS) into a low siMS group (LMS; siMS < 2.085, *n* = 25) and high siMS group (HMS; siMS ≥ 2.085, *n* = 26) groups based on the median siMS score of 2.085 to enable comparisons across study variables. Written informed consent was obtained from all participants, and the study was approved by the Skidmore College Institutional Review Board (IRB#2209 − 1044) in accordance with the Declaration of Helsinki.

### Procedures

This study utilized a cross-sectional, single-visit design to collect data on anthropometrics, body composition, hemodynamics, heart rate variability (HRV), microvascular function, blood glucose and lipid profiles, and dietary intake. All measurements were conducted in a laboratory setting. To standardize conditions, participants were instructed to avoid strenuous exercise for 24 h, refrain from caffeine and alcohol for 12 h, and arrive well-hydrated (consuming 8–12 oz of water the night before and prior to testing).

The methodology for anthropometrics, body composition, hemodynamics, HRV, microvascular function, blood glucose and lipid profiles, and dietary intake followed the exact protocols described in Lang et al. [5], with adjustments to accommodate the LMS and HMS stratification based on siMS scores. Specific hemodynamic measures included central systolic blood pressure (SBP) and diastolic blood pressure (DBP), peripheral SBP and DBP, mean arterial pressure, heart rate, pulse pressure, augmentation pressure, augmentation index (Aix), and Aix adjusted to 75 bpm. HRV measures comprised time-domain variables (percentage of NN intervals differing by more than 50 ms and root mean square of successive differences) and frequency-domain variables (high-frequency power, low-frequency power, and total power). Microvascular reactivity measures included measures of reperfusion, namely the slope 2 or reperfusion rate over 10s, time to peak, and time to halfway, as done previously [31–33].

### SiMS score & risk score

The siMS score was calculated using five metabolic syndrome (MetS) risk factors (waist circumference, triglycerides, high-density lipoprotein cholesterol, peripheral SBP, and blood glucose) following established methods [11, 12]. Specifically, each component was standardized and weighted to compute a continuous score reflecting the severity of cardiometabolic risk. The siMS risk score was derived by incorporating the siMS score, age, and family history risk factors as defined by the International Diabetes Federation and the American Heart Association [11, 12, 34]. These scores offer a simplified and accurate approach for evaluating MetS risk over time, particularly in younger adults without overt MetS [11, 12].

### Statistical analysis

Independent samples t-tests were used to compare the LMS and HMS groups across anthropometrics, body composition, hemodynamics, HRV, microvascular function, blood glucose and lipid profiles, dietary intake, and siMS score and risk score. The number of tests per category ranged from 2 to 10, and a Bonferroni correction was applied within each category to control the family-wise error rate. The alpha level was set at 0.05 per category and divided by the number of tests in that category, yielding adjusted alpha levels from 0.005 (10 tests) to 0.025 (2 tests). This per-category adjustment was chosen to explore differences within distinct physiological domains independently. Levene’s test assessed equality of variances, and Welch’s t-tests were used when variances were unequal, with such instances indicated by a superscripted “a” (within tables) and reported accordingly. Effect sizes were calculated as Cohen’s *d* to quantify the magnitude of differences. Data are reported as means ± SD, unless noted otherwise.

To address potential confounding by demographic factors, multivariable linear regression models were performed for key outcomes showing significant group differences in the t-tests (e.g., body composition variables beyond waist circumference, central blood pressures, and HRV parameters). Each model included the binary siMS group variable (0 = LMS, 1 = HMS) as the primary predictor, with age (continuous) and sex (categorical) entered as covariates. The individual components of the siMS score (waist circumference, triglycerides, HDL cholesterol, peripheral SBP, and fasting glucose) and closely related measures (e.g., body mass index) were not entered as dependent variables in these regression analyses, nor were they included as covariates. Such analyses would be circular and uninformative for the components themselves and would introduce overadjustment bias when examining other outcomes, as these variables either directly constitute the siMS score or lie on the causal pathway between higher cardiometabolic burden and the outcomes of interest.

## Results

### Participants & anthropometrics

All 51 participants were included in the final analyses, comprising 25 in the low Simple Method for Quantifying Metabolic Syndrome (siMS) group (LMS; 56% female, *n* = 14 females and 11 males) and 26 in the high siMS group (HMS; 50% female, *n* = 13 females and 13 males). A chi-square test of independence confirmed no significant association between sex and group allocation, χ²(1) = 0.184, *p* = 0.668, indicating balanced sex distribution across groups. Using an adjusted alpha level of 0.008 for multiple comparisons, the HMS group exhibited significantly higher body mass, body mass index, and waist and hip circumference compared to the LMS group (all *p* < 0.001, *d* = -1.007 to -1.323), indicating distinct anthropometric profiles. No significant differences were observed for age or height under the adjusted alpha level. Detailed values and statistical results are provided in Table 1.

**Table 1:** Anthropometric and demographic characteristics of LMS and HMS groups

Variables	Total (*n* = 51)	LMS (*n* = 25)	HMS (*n* = 26)	*p* Value	ES d
Age (years)	30.5 ± 14.8	25.4 ± 12.0	35.3 ± 15.7	^a^ 0.014	-0.298
Height (cm)	171.3 ± 10.5	170.2 ± 10.0	172.3 ± 11.2	0.474	-0.202
Body mass (kg)	74.7 ± 18.7	66.1 ± 10.5	83.0 ± 21.2	^a^ < 0.001	-1.007
BMI (kg/m^2^)	25.3 ± 4.7	22.7 ± 2.2	27.7 ± 5.1	^a^ < 0.001	-1.279
Waist C (cm)	79.3 ± 14.5	71.9 ± 6.9	86.4 ± 16.3	^a^ < 0.001	-1.161
Hip C (cm)	99.4 ± 10.5	93.4 ± 6.8	105.0 ± 10.4	^a^ < 0.001	-1.323

Data are presented as mean ± standard deviation. Alpha level adjusted for 6 tests using Bonferroni correction (*α* = 0.05/6 = 0.008). Abbreviations: *BMI* body mass index, *C* circumference, *LMS* low siMS score, *HMS* high siMS score

### Body composition

Using an adjusted alpha level of 0.010 for multiple comparisons, the HMS group exhibited significantly higher fat mass, body fat percentage, and visceral fat levels compared to the LMS group (all *p* ≤ 0.003, *d* = -0.306 to -1.116), indicating distinct body composition profiles. No significant differences were observed for fat-free mass or bone mass under the adjusted alpha level. Detailed values and statistical results are provided in Table 2.

**Table 2:** Body composition profiles of LMS and HMS groups

Variables	Total (*n* = 51)	LMS (*n* = 25)	HMS (*n* = 26)	*p* Value	ES d
Fat mass (kg)	19.9 ± 10.4	14.7 ± 4.9	24.8 ± 11.8	^a^ < 0.001	-1.116
Fat-free mass (kg)	54.8 ± 11.8	51.4 ± 9.4	58.1 ± 13.0	0.039	-0.593
Body fat (%)	25.8 ± 8.5	22.3 ± 6.7	29.1 ± 8.8	^a^ 0.003	0.306
Visceral fat (level)	4.9 ± 4.6	2.7 ± 2.2	7.0 ± 5.4	^a^ < 0.001	-1.056
Bone mass (kg)	3.1 ± 1.7	2.6 ± 0.5	3.6 ± 2.2	^a^ 0.025	-0.656

Data are presented as mean ± standard deviation. Alpha level adjusted for 5 tests using Bonferroni correction (*α* = 0.05/5 = 0.010). Abbreviations: *LMS* low siMS score, *HMS* high siMS score

### Hemodynamics

**Fig. 1:**
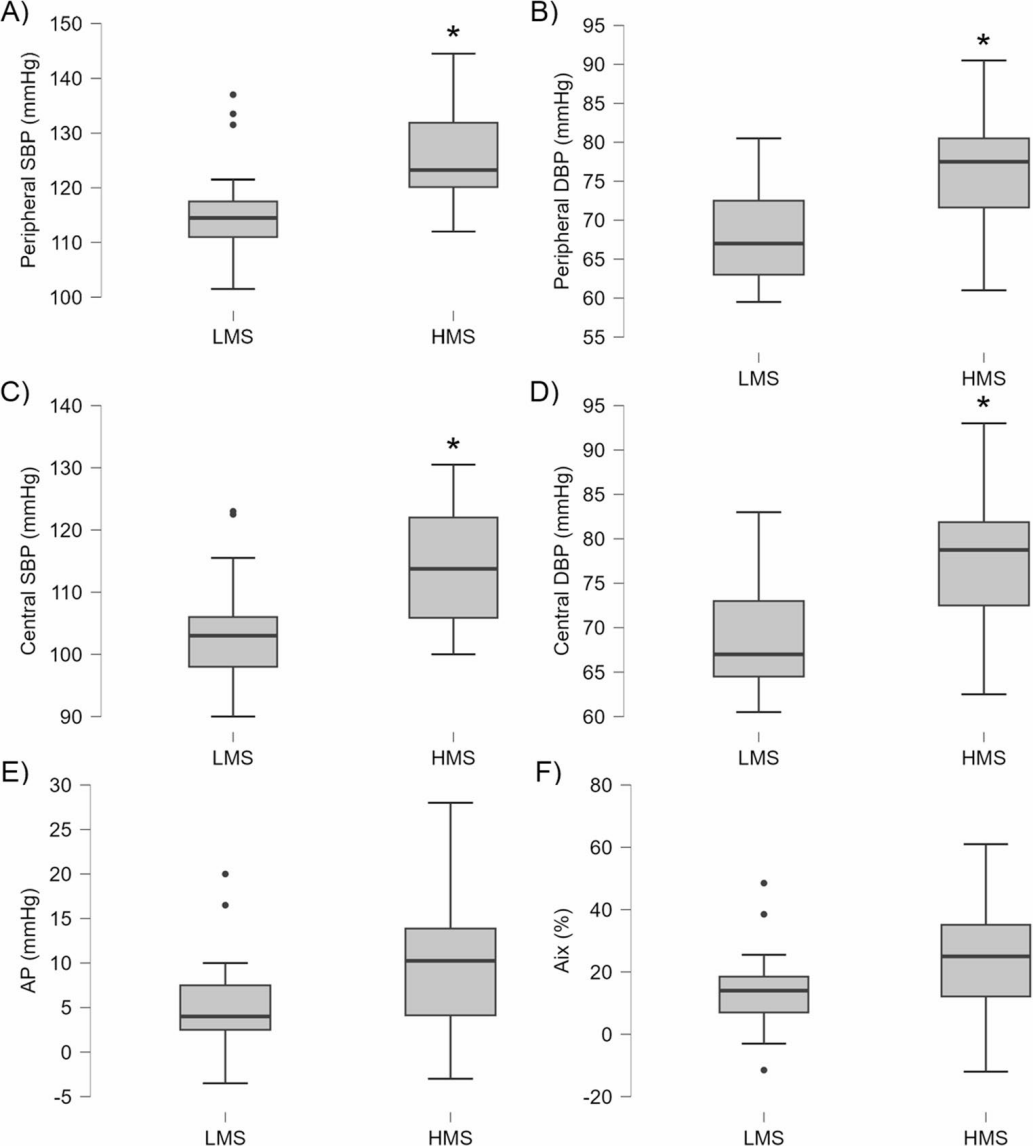
Boxplots showing hemodynamic parameters in LMS and HMS groups. (**A**) Peripheral SBP, (**B**) peripheral DBP, (**C**) central SBP, (**D**) central DBP, (**E**) AP, and (**F**) Aix. * Significantly higher in HMS compared to LMS (*p* < 0.001). Abbreviations: SBP, systolic blood pressure; DBP, diastolic blood pressure; AP, augmentation pressure; Aix, augmentation index; LMS, low siMS score; HMS, high siMS score

Using an adjusted alpha level of 0.005 for multiple comparisons, the HMS group exhibited significantly higher central systolic and diastolic blood pressure (SBP and DBP), peripheral SBP and DBP, mean arterial pressure, and augmentation index adjusted to 75 bpm (Aix 75) compared to the LMS group (all *p* ≤ 0.004, *d* = -0.845 to -1.387), indicating distinct hemodynamic profiles. No significant differences were observed for heart rate, pulse pressure, augmentation pressure, or Aix, under the adjusted alpha level. Detailed values and statistical results are provided in Table 3 with boxplots in Fig. 1.

**Table 3:** Hemodynamic parameters of LMS and HMS groups

Variables	Total (*n* = 51)	LMS (*n* = 25)	HMS (*n* = 26)	*p* Value	ES d
HR (bpm)	62.0 ± 10.1	59.4 ± 10.1	64.4 ± 9.7	0.079	-0.502
C SBP (mmHg)	108.7 ± 10.1	103.0 ± 8.3	114.1 ± 8.7	< 0.001	-1.299
C DBP (mmHg)	73.2 ± 7.8	69.2 ± 6.1	77.0 ± 7.4	< 0.001	-1.149
P SBP (mmHg)	120.8 ± 9.6	115.6 ± 8.5	125.9 ± 7.8	< 0.001	-1.263
P DBP (mmHg)	72.3 ± 7.7	68.3 ± 6.0	76.1 ± 7.2	< 0.001	-1.179
PP (mmHg)	35.4 ± 6.3	33.6 ± 7.4	37.1 ± 4.4	^a^ 0.045	-0.581
MAP (mmHg)	87.1 ± 8.9	81.9 ± 6.2	92.1 ± 8.3	< 0.001	-1.387
AP (mmHg)	7.6 ± 6.7	5.2 ± 5.1	9.9 ± 7.4	0.011	-0.744
Aix (%)	19.7 ± 16.0	14.2 ± 12.4	25.0 ± 17.3	0.013	-0.718
Aix 75 (%)	13.6 ± 16.2	7.1 ± 13.6	19.9 ± 16.3	0.004	-0.845

Data are presented as mean ± standard deviation. Alpha level adjusted for 10 tests using Bonferroni correction (*α* = 0.05/10 = 0.005). Abbreviations: *HR* heart rate, *C* central, *SBP* systolic blood pressure, *DBP* diastolic blood pressure, *P* peripheral, *PP* pulse pressure, *MAP* mean arterial pressure, *AP* augmentation pressure, *Aix* augmentation index, *LMS* low siMS score, *HMS* high siMS score

### Heart rate variability metrics

**Fig. 2:**
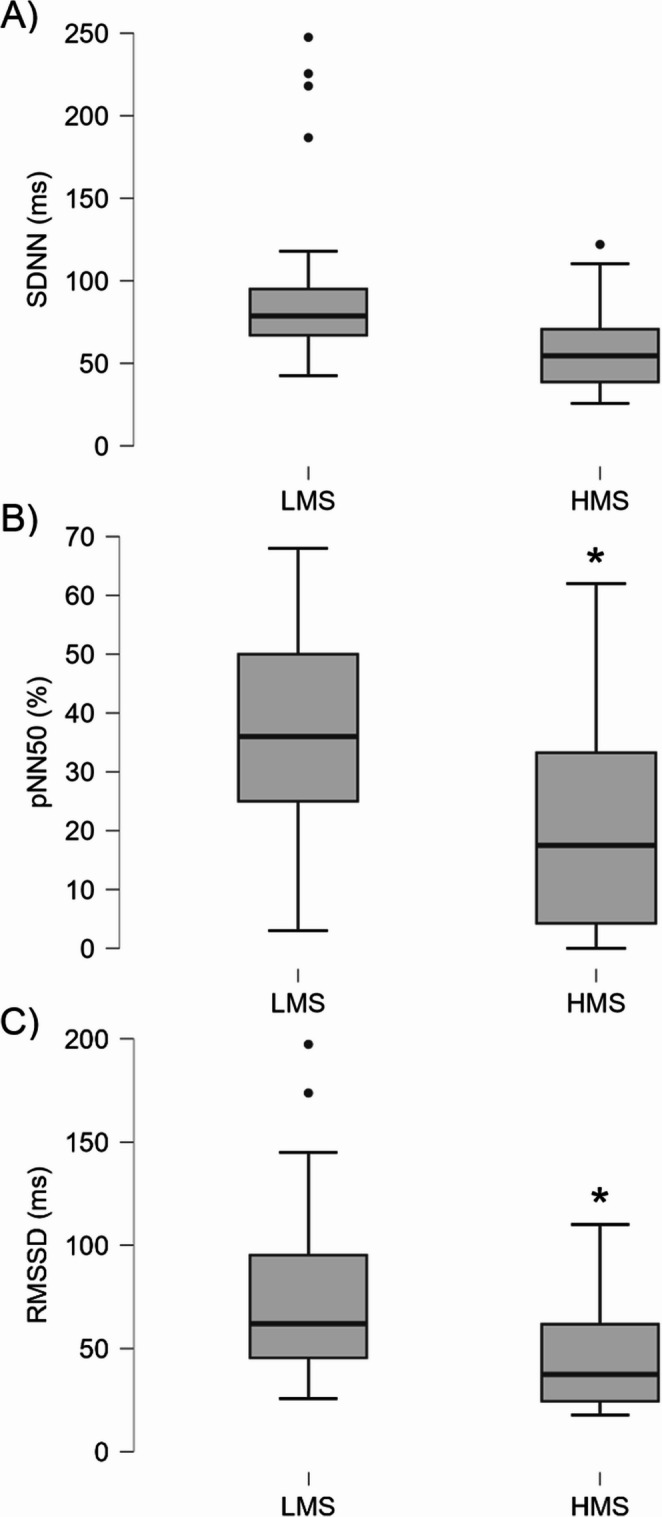
Boxplots showing heart rate variability parameters in LMS and HMS groups. (**A**) SDNN, (**B**) pNN50, and (**C**) RMSSD. * Significantly lower in HMS compared to LMS (*p* ≤ 0.003). Abbreviations: SDNN, standard deviation of normal-to-normal intervals; pNN50, percentage of NN intervals differing by more than 50 ms; RMSSD, root mean square of successive differences; LMS, low siMS score; HMS, high siMS score

Using an adjusted alpha level of 0.008 for multiple comparisons, the HMS group exhibited significantly lower percentage of normal-to-normal intervals differing by more than 50 ms, root mean square of successive differences and frequency-domain variables (high-frequency power, low-frequency power, and total power) compared to the LMS group (all *p* ≤ 0.003, *d* = -0.884 to -1.323), indicating distinct autonomic function profiles. No significant differences were observed for standard deviation of normal-to-normal intervals under the adjusted alpha level. Detailed values and statistical results are provided in Table 4 with boxplots in Fig. 2.

**Table 4:** Heart rate variability metrics of LMS and HMS groups

Variables	Total (*n* = 51)	LMS (*n* = 25)	HMS (*n* = 26)	*p* Value	ES d
SDNN (ms)	78.2 ± 47.6	99.1 ± 56.8	58.2 ± 24.3	^a^ 0.014	-0.298
pNN50 (%)	29.6 ± 20.5	38.2 ± 18.5	21.3 ± 19.1	< 0.001	-1.323
RMSSD (ms)	61.4 ± 38.9	77.8 ± 44.2	45.8 ± 25.2	^a^ 0.003	-0.884
HF Power (ms^2^)	3790.6 ± 12089.2	6595.3 ± 16933.9	1093.8 ± 1257.9	^a^ < 0.001	-1.007
LF Power (ms^2^)	3470.0 ± 7666.6	5852.0 ± 10458.5	1179.7 ± 1191.5	^a^ < 0.001	-1.279
Total Power (ms^2^)	7260.6 ± 19479.6	12445.4 ± 27021.8	2275.3 ± 2281.8	^a^ < 0.001	-1.161

Data are presented as mean ± standard deviation. Alpha level adjusted for 6 tests using Bonferroni correction (*α* = 0.05/6 = 0.008). Abbreviations: *SDNN* standard deviation of normal-to-normal intervals, *pNN50* percentage of NN intervals differing by more than 50 ms, *RMSSD* root mean square of successive differences, *HF* high-frequency, *LF* low-frequency, *LMS* low siMS score, *HMS* high siMS score

### Microvascular reactivity

Using an adjusted alpha level of 0.0167 for multiple comparisons, no significant differences were observed between the LMS and HMS groups for Slope 2 F (10s), Slope 2 F (time to peak), or Slope 2 F (time to halfway) (all *p* > 0.0167). Detailed values and statistical results are provided in Table 5.

**Table 5:** Microvascular reactivity of LMS and HMS groups

Variables	Total (*n* = 51)	LMS (*n* = 25)	HMS (*n* = 26)	*p* Value	ES d
Slope 2 (10s)	2.09 ± 1.39	2.03 ± 1.28	2.13 ± 1.51	0.795	-0.073
Slope 2 (time to peak)	57.5 ± 24.7	61.0 ± 27.6	54.1 ± 21.6	0.326	0.278
Slope 2 (time to halfway)	28.1 ± 24.4	32.4 ± 28.3	23.9 ± 19.6	0.221	0.347

Data are presented as mean ± standard deviation. Alpha level adjusted for 3tests using Bonferroni correction (*α* = 0.05/3 = 0.0167). Abbreviations: *LMS* low siMS score, *HMS* high siMS score

### Blood glucose and lipid profiles

Using an adjusted alpha level of 0.007 for multiple comparisons, no significant differences were observed between the LMS and HMS groups for triglycerides (TG), blood glucose, total cholesterol, high-density lipoprotein (HDL) cholesterol, low-density lipoprotein cholesterol, non-HDL cholesterol, or total cholesterol-to-HDL ratio (all *p* > 0.007). Detailed values and statistical results are provided in Table 6.

**Table 6:** Blood glucose and lipid profiles of LMS and HMS groups

Variables	Total (*n* = 51)	LMS (*n* = 25)	HMS (*n* = 26)	*p* Value	ES d
TG (mmol/L)	0.91 ± 0.48	0.93 ± 0.50	0.88 ± 0.46	0.738	0.094
BG (mmol/L)	4.84 ± 0.43	4.80 ± 0.40	4.87 ± 0.47	0.527	-0.178
TC (mmol/L)	4.14 ± 0.82	4.19 ± 0.96	4.10 ± 0.67	^a^ 0.708	0.106
HDL C (mmol/L)	1.29 ± 0.36	1.34 ± 0.38	1.23 ± 0.34	0.262	0.318
LDL C (mmol/L)	2.45 ± 0.75	2.42 ± 0.86	2.47 ± 0.65	0.834	-0.059
Non-HDL C (mmol/L)	2.86 ± 0.89	2.85 ± 0.98	2.87 ± 0.81	0.921	-0.028
TC: HDL (#)	3.50 ± 1.3	3.34 ± 1.05	3.65 ± 1.42	0.381	-0.248

Data are presented as mean ± standard deviation. Alpha level adjusted for 7 tests using Bonferroni correction (*α* = 0.05/7 = 0.007). Abbreviations: *TG* triglyceride, *BG* blood glucose, *TC* total cholesterol, *HDL* high-density lipoprotein, *LDL* low-density lipoprotein, *LMS* low siMS score, *HMS* high siMS score

### SiMS score & risk score and MetS risk

For siMS score, a significant difference (t_49_ = -7.959, *p* < 0.001, *d* = -2.229) between LMS and HMS was observed, with the HMS group showing a higher siMS score (mean ± SD: 2.586 ± 0.489) compared to the LMS group (mean ± SD: 1.671 ± 0.306). For siMS risk score, a significant difference (Welch’s t_33.506_ = -4.781, *p* < 0.001, *d* = -1.330) between LMS and HMS was observed, with the HMS group showing a high siMS risk score (mean ± SD: 2.019 ± 1.108) compared to the LMS group (mean ± SD: 0.892 ± 0.456). Total siMS score and siMS risk score (i.e., for all participants) were 2.137 ± 0.615 and 1.466 ± 1.018, respectively (mean ± SD).

In the LMS group, 90% (9/10) of participants with MetS risk factors had one factor (8 with low HDL, 1 with high SBP), and 10% (1/10) had two (high SBP and TG). Conversely, the HMS group’s risk factor distribution showed 58.8% (10/17) with one factor (6 with low HDL, 4 with high SBP), 23.5% (4/17) with two (e.g., low HDL and high SBP), 11.8% (2/17) with three (e.g., high waist circumference, high SBP, low HDL), and 5.9% (1/17) with four (high waist circumference, high SBP, high TG, low HDL).

### Dietary intake

Using an adjusted alpha level of 0.005 for multiple comparisons, no significant differences were observed between the LMS and HMS groups for absolute or relative intakes of energy, carbohydrates, lipids, proteins, or fiber (all *p* > 0.005). Detailed values and statistical results are provided in Table 7.

**Table 7:** Dietary energy, macronutrient, and fiber intake of LMS and HMS groups

Variables		Total (*n* = 51)	LMS (*n* = 25)	HMS (*n* = 26)	*p* Value	ES d
Energy (kcal)	A	2218 ± 1089	2186 ± 944	2249 ± 1231	0.838	-0.057
	R	31.0 ± 16.0	33.6 ± 15.3	28.5 ± 16.6	0.264	0.317
Carbs (g)	A	238 ± 130	222 ± 117	253 ± 143	0.406	-0.235
	R	3.4 ± 2.0	3.8 ± 2.5	2.9 ± 1.4	^a^ 0.101	0.474
Lipids (g)	A	95 ± 50	98 ± 46	93 ± 55	0.707	0.106
	R	1.3 ± 0.7	1.5 ± 0.7	1.2 ± 0.7	0.105	0.463
Proteins (g)	A	104 ± 64	105 ± 56	103 ± 72	0.914	0.030
	R	1.4 ± 0.9	1.6 ± 0.8	1.3 ± 1.0	0.267	0.315
Fiber (g)	A	21 ± 14	21 ± 11	22 ± 16	0.965	-0.012

Data are presented as mean ± standard deviation. Alpha level adjusted for 7 tests using Bonferroni correction (*α* = 0.05/9 = 0.005). Abbreviations: *Carbs* carbohydrates, *A* absolute intake (/d), *R* relative intake (/kg/d), *LMS* low siMS score, *HMS* high siMS score

### Multivariable regression analyses

Multivariable linear regression models adjusted for age and sex largely confirmed the unadjusted differences between the HMS and LMS groups across key cardiometabolic and autonomic outcomes (Table 8). Higher siMS burden was independently associated with greater body mass (β = 14.50 kg), larger hip circumference (β = 9.21 cm), higher fat mass (β = 9.08 kg), elevated visceral fat level (β = 2.29), and increased central SBP (β = 7.96 mmHg), central DBP (β = 6.39 mmHg), peripheral DBP (β = 6.31 mmHg), and MAP (β = 8.60 mmHg; all *p* ≤ 0.002 for group variable). Reductions in HRV parameters (pNN50, high-frequency power, low-frequency power, and total power) remained directionally consistent with poorer autonomic function in the HMS group, although the overall models for power variables did not reach statistical significance (*p* = 0.012–0.033 for group; model *p* ≈ 0.080). The previously observed difference in Aix 75 was no longer significant (*p* = 0.067), with age emerging as the primary predictor.

**Table 8:** Model summaries of separate multiple linear regression models and coefficients for key outcomes when controlling for age, sex (female and male), and group (HMS and LMS)

Outcome	β ± SE (group)	*p* (group)	*R* ^2^	Adj *R*^2^	*p* (model)	Sig predictors
Body mass (kg)	14.503 ± 4.344	0.002	0.431	0.395	< 0.001	sex, group
Hip cir (cm)	9.208 ± 2.399	< 0.001	0.447	0.412	< 0.001	age, sex, group
Fat mass (kg)	9.079 ± 2.741	0.002	0.262	0.215	0.002	group
Visceral fat (level)	2.289 ± 0.850	0.010	0.643	0.620	< 0.001	age, sex, group
C SBP (mmHg)	7.963 ± 2.230	< 0.001	0.485	0.452	< 0.001	age, group
C DBP (mmHg)	6.388 ± 1.944	0.002	0.341	0.299	< 0.001	age, group
P DBP (mmHg)	6.306 ± 1.864	0.001	0.373	0.333	< 0.001	age, group
MAP (mmHg)	8.596 ± 2.110	< 0.001	0.410	0.372	< 0.001	age, group
Aix 75 (%)	6.922 ± 3.696	0.067	0.452	0.417	< 0.001	age
pNN50 (%)	-11.656 ± 5.265	0.032	0.302	0.257	< 0.001	sex, group
HF power (ms^2^)	-7604.7 ± 3462.7	0.033	0.132	0.077	0.080	group
LF power (ms^2^)	-5738.7 ± 2192.9	0.012	0.135	0.080	0.076	group
Total power (ms^2^)	-13340.0 ± 5577.6	0.021	0.133	0.078	0.079	group

Data are presented as mean ± standard deviation. No additional correction to the alpha level (*α* = 0.05) for multiple comparisons was applied to these confirmatory models. Abbreviations: *β* beta, *SE* standard error, *Sig* significant, *cir* circumference, *C* central, *SBP* systolic blood pressure, *DBP* diastolic blood pressure, *P* peripheral, *MAP* mean arterial pressure, *Aix* augmentation index, *pNN50* percentage of NN intervals differing by more than 50 ms, *RMSSD* root mean square of successive differences, *HF* high-frequency, *LF* low-frequency, *LMS* low siMS score, *HMS* high siMS score

## Discussion

This study compared cardiometabolic burden in younger adults stratified by the Simple Method for Quantifying Metabolic Syndrome (siMS) score, focusing on central hemodynamic parameters, heart rate variability (HRV), and microvascular function as indicators of cardiovascular disease (CVD) risk. The high siMS (HMS) group exhibited significantly higher siMS scores and risk scores, reflecting a greater prevalence of metabolic syndrome (MetS) risk factors. Moreover, independent risk factors such as HRV showed reduction in parasympathetic nervous system activity (both time and frequency domain), as well as elevated central blood pressure, in this relatively low siMS score cohort. These findings appear relatively independent of differences in dietary intakes, which were not different between groups. Future studies and clinicians should consider a broader evaluative framework in those being monitored for MetS risk.

### Anthropometric and body composition contributions to siMS scores

The HMS group showed adiposity, including higher body mass, body mass index, waist and hip circumferences (Table 1), and elevated fat mass, body fat percentage, and visceral fat (Table 2), reflecting the siMS score’s emphasis on waist circumference as a key MetS component [6, 11]. These findings, with large effect sizes (*d* = -1.007 to -1.323), underscore adiposity’s role in early cardiometabolic risk [7, 35–37]. Multivariable regression analyses adjusting for age and sex confirmed that higher siMS burden remained independently associated with greater body mass, hip circumference, fat mass, and visceral fat level (all *p* ≤ 0.010), reinforcing the central role of adiposity in driving siMS scores and early cardiometabolic risk, beyond simple demographic influences. Yet only 5 participants, all in HMS, met the MetS criteria for waist circumference. This limited prevalence highlights the siMS score’s sensitivity to anthropometric factors in a young cohort (mean ~ 30.5 years), supporting its utility for detecting preclinical risk [11, 12]. Though it remains to be seen if assessments of visceral adipose prove superior to waist circumference or waist-to-hip ratio.

### Hemodynamic and autonomic dysfunction implications and CVD risk

The HMS group exhibited higher central and peripheral systolic and diastolic blood pressures (SBP and DBP), mean arterial pressure (MAP), and augmentation index adjusted to 75 bpm (Aix 75; Table 3), which may indicate increased cardiovascular load, consistent with central hemodynamics linked to CVD risk in prior studies [13, 15, 16, 18, 19]. The lack of significant differences in augmentation pressure and Aix under the adjusted alpha (0.005) suggests that pulse wave augmentation may be less pronounced in this cohort [17]. The higher waist circumference and visceral fat levels in HMS likely contributed to these hemodynamic changes by increasing vascular resistance and sympathetic activity [23, 26–28]. After adjustment for age and sex, higher siMS burden continued to show strong independent associations with elevated central SBP and DBP, peripheral DBP, and MAP (all *p* ≤ 0.002), supporting a direct link between cardiometabolic burden and increased cardiovascular load in this young cohort. In contrast, the association with Aix 75 was attenuated and no longer significant after adjustment (*p* = 0.067), suggesting that age largely explains this difference.

These elevations in cardiovascular load and suggested increases in sympathetic tone may also influence autonomic nervous system balance. This may be reflected in the low siMS (LMS) group’s higher HRV metrics (percentage of NN intervals differing by more than 50 ms [pNN60], high-frequency power, low-frequency power, and total power; Table 4), indicating greater parasympathetic activity compared to the HMS group, where lower HRV suggests potential autonomic differences [23–25]. Reductions in HRV parameters (pNN50, high-frequency, low-frequency, and total power) remained directionally consistent with poorer autonomic function in the HMS group using linear regression (*p* = 0.012–0.033), though the models themselves did not reach statistical significance. This pattern aligns with the hypothesis of early autonomic impairment but highlights the need for larger samples to confirm these trends given the known variability in HRV measures.

The lower HRV metrics in HMS may contribute to CVD risk by impairing heart rate and blood pressure regulation, as supported by previous research [21–25]. The absence of significant differences in Slope 2 from the near-infrared spectroscopy vascular occlusion test (Table 5) suggests that microvascular reactivity does not vary with siMS score in this cohort, potentially indicating that microvascular dysfunction may not be an early marker of MetS-related risk in this young population [29, 30]. These findings suggest that higher siMS scores are associated with reduced parasympathetic activity as indicated by HRV metrics, potentially increasing BP and/or CVD risk.

### Unexpected findings: blood glucose, lipid profiles, and dietary intake

No differences were observed in blood glucose, lipid profiles, or dietary intake between LMS and HMS groups (Tables 5 and 6). Notably, 8 participants in the LMS group and 11 in the HMS group had low high-density lipoprotein (HDL), indicating a prevalent MetS risk factor in both groups despite the lack of statistical difference in overall lipid profiles. This is unexpected given the siMS score’s inclusion of triglycerides, HDL, and glucose, but may reflect the young age of the cohort (mean ~ 30.5 years), where metabolic abnormalities are not yet pronounced [6, 8, 10, 37]. The lack of dietary differences suggests that other factors, such as physical activity or energy balance, rather than absolute energy intakes, may drive the HMS group’s risk profile [6, 10, 35, 36]. Similarly, the lack of difference in Slope 2 reinforces the notation that the HMS group’s risk profile may not involve detectable microvascular changes, aligning with the absence of metabolic abnormalities [29, 30].

### Implications and integration with siMS score

Higher siMS scores in the HMS group were associated with greater anthropometric, hemodynamic, and lower HRV metrics, despite no differences in metabolic, dietary, or microvascular markers, highlighting the siMS score’s ability to detect early cardiometabolic burden, even in pre-MetS individuals. For reference, Šebeková et al. reported that higher siMS quintiles (3Q-5Q) had 28.7% to 36.1% of participants with one MetS component and 11.8% to 51.5% with two, while only 11.5% (3/26) of our HMS participants met the MetS diagnostic criteria (three or more factors), compared to 28.4% in their 5Q, indicating an intermediate risk profile [12]. The HMS group’s intermediate risk profile, with only 11.5% meeting MetS criteria compared to 28.4% in Šebeková et al.’s 5Q, underscores the siMS score’s potential for early risk stratification [12]. Importantly, the persistence of significant associations for anthropometric, body composition, and central hemodynamic outcomes after controlling for age and sex (Table 8) indicates that higher siMS burden captures cardiometabolic risk that is not merely attributable to demographic differences between groups. This strengthens confidence in the siMS score as a tool for early risk identification in young adults, even when traditional metabolic markers (glucose, lipids) remain unremarkable.

### Limitations

This study has several limitations. First, some participants’ triglyceride values (*n* = 16) were below the minimal acceptable threshold (45 mg/dL) of the Cholestech LDX analyzer. To maintain data consistency and sample size, these values were set to the minimal acceptable threshold [10]. Second, cross-sectional design limits causal inferences about the relationship between siMS scores, hemodynamics, and HRV, as well as microvascular function, necessitating longitudinal studies to confirm these associations. Third, the relatively small sample size (*n* = 51) and focus on a relatively healthy, younger cohort may limit generalizability to older or more clinically diverse populations. Additionally, detailed demographic data beyond sex (e.g., race/ethnicity, socioeconomic status) were not included, further restricting our ability to evaluate the diversity of the sample and the broader applicability of the findings. Future studies should include larger, more diverse samples to enhance generalizability.

## Conclusion

In conclusion, this study demonstrates that higher siMS scores are associated with poorer anthropometric measures, hemodynamic differences, and lower HRV, reflecting early cardiometabolic risk even in the absence of overt MetS or microvascular and metabolic abnormalities. The siMS score may serve as a proactive tool for identifying at-risk individuals, supporting targeted interventions to mitigate long-term CVD risk in younger populations.

## Data Availability

The datasets used and analyzed during the current study are available from the corresponding author on reasonable request.

## Declarations

### Clinical trial number

Trial registered with ClinicalTrials.gov (NCT06544915, Registration Date: 08/06/2024).

## References

[1] Chee Cheong K, Lim KH, Ghazali SM, Teh CH, Cheah YK, Baharudin A, et al. Association of metabolic syndrome with risk of cardiovascular disease mortality and all-cause mortality among Malaysian adults: a retrospective cohort study. BMJ Open. 2021;11:e047849. 10.1136/bmjopen-2020-047849. 34408040 10.1136/bmjopen-2020-047849 PMC8375738

[2] Hirode G, Wong RJ. Trends in the prevalence of metabolic syndrome in the united States, 2011–2016. JAMA. 2020;323:2526. 10.1001/jama.2020.4501. 32573660 10.1001/jama.2020.4501 PMC7312413

[3] Malik S, Wong ND, Franklin SS, Kamath TV, L’Italien GJ, Pio JR, et al. Impact of the metabolic syndrome on mortality from coronary heart disease, cardiovascular disease, and all causes in united States adults. Circulation. 2004;110:1245–50. 10.1161/01.CIR.0000140677.20606.0E. 15326067 10.1161/01.CIR.0000140677.20606.0E

[4] Rus M, Crisan S, Andronie-Cioara FL, Indries M, Marian P, Pobirci OL, et al. Prevalence and risk factors of metabolic syndrome: A prospective study on cardiovascular health. Med (Lithuania). 2023;59. 10.3390/medicina59101711. 10.3390/medicina59101711 PMC10608643 37893429

[5] Lang JM, Shostak ES, Quinn WK, Chervinskaya VD, Fioraso E, Smith E, et al. Dyslipidemia impacts cardiometabolic health and CVD risk in a relatively young otherwise healthy population. J Clin Hypertens. 2025;27. 10.1111/jch.14972. 10.1111/jch.14972 PMC11771811 39821451

[6] Sawyer B, Stone KA, Kotarsky CJ, Johnson N, Bradley A, Scheffert RA, et al. Animal-Based dietary protein intake is not A risk factor for metabolic syndrome among young or Middle-Aged females. Nutr Metab Insights. 2022;15. 10.1177/11786388221107800. 10.1177/11786388221107800 PMC9234837 35769392

[7] Ying M, Hu X, Li Q, Dong H, Zhou Y, Chen Z. Long-term trajectories of BMI and cumulative incident metabolic syndrome: A cohort study. Front Endocrinol (Lausanne). 2022;13. 10.3389/fendo.2022.915394. 10.3389/fendo.2022.915394 PMC9773063 36568079

[8] Poon VTW, Kuk JL, Ardern CI. Trajectories of metabolic syndrome development in young adults. PLoS ONE. 2014;9:e111647. 10.1371/journal.pone.0111647. 25368999 10.1371/journal.pone.0111647 PMC4219745

[9] Franco OH, Massaro JM, Civil J, Cobain MR, O’Malley B, D’Agostino RB. Trajectories of entering the metabolic syndrome: the Framingham heart study. Circulation. 2009;120:1943–50. 10.1161/CIRCULATIONAHA.109.855817. 19884471 10.1161/CIRCULATIONAHA.109.855817

[10] Kotarsky CJ, Frenett ML, Hoerle WF, Kim J, Lockwood J, Cryer L et al. Plant-Based dietary protein is associated with lower metabolic syndrome risk in division III female athletes: A pilot study. Nutrients. 2024;16. 10.3390/nu16203486 10.3390/nu16203486 PMC11510158 39458481

[11] Soldatovic I, Vukovic R, Culafic D, Gajic M, Dimitrijevic-Sreckovic V. SiMS score: simple method for quantifying metabolic syndrome. PLoS ONE. 2016;11:1–10. 10.1371/journal.pone.0146143. 10.1371/journal.pone.0146143 PMC4706421 26745635

[12] Sebekova K, Sebek J. Continuous metabolic syndrome score (siMS) enables quantifi cation of severity of cardiometabolic affliction in individuals not presenting with metabolic syndrome. Bratislava Med J. 2018;119:675–8. 10.4149/BLL_2018_121. 10.4149/BLL_2018_121 30672711

[13] Cheng YB, Li Y, Cheng HM, Siddique S, Huynh M, Van, Sukonthasarn A, et al. Central hypertension is a non-negligible cardiovascular risk factor. J Clin Hypertens. 2022;24:1174–9. 10.1111/jch.14561. 10.1111/jch.14561 PMC9532928 36196474

[14] Cheng HM, Chuang SY, Sung SH, Yu WC, Pearson A, Lakatta EG, et al. Derivation and validation of diagnostic thresholds for central blood pressure measurements based on long-term cardiovascular risks. J Am Coll Cardiol. 2013;62:1780–7. 10.1016/j.jacc.2013.06.029. 23850921 10.1016/j.jacc.2013.06.029 PMC3884552

[15] Dong Y, Jiang L, Wang X, Chen Z, Zhang L, Zhang Z, et al. Central rather than brachial pressures are stronger predictors of cardiovascular outcomes: A longitudinal prospective study in a Chinese population. J Clin Hypertens. 2020;22:623–30. 10.1111/jch.13838. 10.1111/jch.13838 PMC8029759 32153115

[16] Agabiti-Rosei E, Mancia G, O’Rourke MF, Roman MJ, Safar ME, Smulyan H, et al. Central blood pressure measurements and antihypertensive therapy: A consensus document. Hypertension. 2007;50:154–60. 10.1161/HYPERTENSIONAHA.107.090068. 17562972 10.1161/HYPERTENSIONAHA.107.090068

[17] Chirinos JA, Zambrano JP, Chakko S, Veerani A, Schob A, Willens HJ, et al. Aortic pressure augmentation predicts adverse cardiovascular events in patients with established coronary artery disease. Hypertension. 2005;45:980–5. 10.1161/01.HYP.0000165025.16381.44. 15837821 10.1161/01.HYP.0000165025.16381.44

[18] Lamarche F, Agharazii M, Madore F, Goupil R. Prediction of cardiovascular events by type I central systolic blood pressure: A prospective study. Hypertension. 2021;77:319–27. 10.1161/HYPERTENSIONAHA.120.16163. 33307853 10.1161/HYPERTENSIONAHA.120.16163 PMC7803443

[19] Kollias A, Lagou S, Zeniodi ME, Boubouchairopoulou N, Stergiou GS. Association of central versus brachial blood pressure with Target-Organ damage: systematic review and Meta-Analysis. Hypertension. 2016;67:183–90. 10.1161/HYPERTENSIONAHA.115.06066. 26597821 10.1161/HYPERTENSIONAHA.115.06066

[20] Laucyte-Cibulskiene A, Chen CH, Cockroft J, Cunha PG, Kavousi M, Laucevicius A, et al. Clusters of risk factors in metabolic syndrome and their influence on central blood pressure in a global study. Sci Rep. 2022;12. 10.1038/s41598-022-18094-y. 10.1038/s41598-022-18094-y PMC9402529 36002468

[21] Buccelletti E, Gilardi E, Scaini E, Galiuto L, Persiani R, Biondi A, et al. Heart rate variability and myocardial infarction: systematic literature review and metanalysis. Eur Rev Med Pharmacol Sci. 2009;13:299–307. 19694345

[22] Salmoirago-Blotcher E, Hovey KM, Andrews CA, Allison M, Brunner RL, Denburg NL, et al. Psychological Traits, heart rate Variability, and risk of coronary heart disease in healthy aging Women - The women’s health initiative. Psychosom Med. 2019;81:256–64. 10.1097/PSY.0000000000000672. 30688770 10.1097/PSY.0000000000000672 PMC6443472

[23] Shah AS, Jaiswal M, Dabelea D, Divers J, Isom S, Liese AD, et al. Cardiovascular risk and heart rate variability in young adults with type 2 diabetes and arterial stiffness: the SEARCH for diabetes in youth study. J Diabetes Complications. 2020;34. 10.1016/j.jdiacomp.2020.107676. 10.1016/j.jdiacomp.2020.107676 PMC7502460 32713707

[24] Hillebrand S, Gast KB, De Mutsert R, Swenne CA, Jukema JW, Middeldorp S, et al. Heart rate variability and first cardiovascular event in populations without known cardiovascular disease: Meta-analysis and dose-response meta-regression. Europace. 2013;15:742–9. 10.1093/europace/eus341. 23370966 10.1093/europace/eus341

[25] Orini M, van Duijvenboden S, Young WJ, Ramírez J, Jones AR, Hughes AD, et al. Long-term association of ultra-short heart rate variability with cardiovascular events. Sci Rep. 2023;13. 10.1038/s41598-023-45988-2. 10.1038/s41598-023-45988-2 PMC10624663 37923787

[26] Williams SM, Eleftheriadou A, Alam U, Cuthbertson DJ, Wilding JPH. Cardiac autonomic neuropathy in Obesity, the metabolic syndrome and prediabetes: A narrative review. Diabetes Therapy. 2019;10:1995–2021. 10.1007/s13300-019-00693-0. 31552598 10.1007/s13300-019-00693-0 PMC6848658

[27] Kazamel M, Stino AM, Smith AG. Metabolic syndrome and peripheral neuropathy. Muscle Nerve. 2021;63:285–93. 10.1002/mus.27086. 33098165 10.1002/mus.27086

[28] Yu TY, Lee MK. Autonomic dysfunction, diabetes and metabolic syndrome. J Diabetes Investig. 2021;12:2108–11. 10.1111/jdi.13691. 34622579 10.1111/jdi.13691 PMC8668070

[29] Soares RN, Murias JM. Near-infrared spectroscopy assessment of microvasculature detects difference in lower limb vascular responsiveness in obese compared to lean individuals. Microvasc Res. 2018;118:31–5. 10.1016/j.mvr.2018.01.008. 29408423 10.1016/j.mvr.2018.01.008

[30] Rogers EM, Banks NF, Jenkins NDM. Metabolic and microvascular function assessed using near-infrared spectroscopy with vascular occlusion in women: age differences and reliability. Exp Physiol. 2023;108:123–34. 10.1113/EP090540. 36420592 10.1113/EP090540 PMC10103776

[31] Shostak ES, Lang JM, Quinn WK, Chervinskaya VD, Fioraso E, Smith E, et al. Effects of selective serotonin reuptake inhibitor (SSRI) use on cardiometabolic health and risk in young healthy individuals: A preliminary matched pairs study. Physiol Rep. 2025;13. 10.14814/phy2.70285. 10.14814/phy2.70285 PMC12032445 40285449

[32] Blum OE, DeBlauw JA, Greaves LM, Shostak ES, Ives SJ. Environmental exposure to wildfire smoke May reduce microvascular oxygenation during graded handgrip exercise: A case series. Physiol Rep. 2024;12. 10.14814/phy2.16120. 10.14814/phy2.16120 PMC11189769 39031617

[33] Zaleski K, Matias A, Gyampo A, Giuriato G, Lynch M, Lora B, et al. Does sex influence near-infrared spectroscopy-derived indicators of microvascular reactivity and the response to acute dietary capsaicin. Microvasc Res. 2023;145. 10.1016/j.mvr.2022.104436. 10.1016/j.mvr.2022.104436 36113667

[34] Alberti KGMM, Eckel RH, Grundy SM, Zimmet PZ, Cleeman JI, Donato KA, et al. Harmonizing the metabolic syndrome: A joint interim statement of the international diabetes federation task force on epidemiology and prevention; National heart, lung, and blood institute; American heart association; world heart federation; international atherosclerosis society; and international association for the study of obesity. Circulation. 2009;120:1640–5. 10.1161/CIRCULATIONAHA.109.192644. 19805654 10.1161/CIRCULATIONAHA.109.192644

[35] Chiang TL, Chen C, Hsu CH, Lin YC, Wu HJ. Is The goal of 12,000 steps per day sufficient for improving body composition and metabolic syndrome? The necessity of combining exercise intensity: A randomized controlled trial. BMC Public Health. 2019;19. 10.1186/s12889-019-7554-y 10.1186/s12889-019-7554-y PMC6724241 31481039

[36] Gallardo-Alfaro L, Del Mar Bibiloni M, Mascaró CM, Montemayor S, Ruiz-Canela M, Salas-Salvad J et al. Leisure-time physical activity, sedentary behaviour and diet quality are associated with metabolic syndrome severity: the PREDIMED-plus study. Nutrients. 2020;12. 10.3390/nu12041013 10.3390/nu12041013 PMC7230557 32272653

[37] Cornier M-A, Dabelea D, Hernandez TL, Lindstrom RC, Steig AJ, Stob NR, et al. Metabolic Syndrome Endocr Rev. 2008;29:777–822. 10.1210/er.2008-0024. 18971485 10.1210/er.2008-0024 PMC5393149

